# Acidosis Drives Vasculogenic Mimicry in PDAC CSCs via Na+/H+ Exchanger Isoform 1 (NHE1) and Calcium Entry

**DOI:** 10.3390/cells15100865

**Published:** 2026-05-09

**Authors:** Maria Raffaella Greco, Francesca Fracasso, Stefania Cannone, Daria Di Molfetta, Marilena Ardone, Sharon Natasha Cox, Brunella Rita Ladogana, Daniela Isabel Abbrescia, Apollonia Tullo, Marianna Ranieri, Stephan J. Reshkin, Rosa Angela Cardone

**Affiliations:** 1 Department of Bioscience, Biotechnologies and Environment, University of Bari Aldo Moro, Via E. Orabona 4, 70126 Bari, Italy; grecoraffaella1975@gmail.com (M.R.G.); francesca.fracasso@uniba.it (F.F.); stefaniacannone92@gmail.com (S.C.); daria.dimolfetta@uniba.it (D.D.M.); marilena.ardone@uniba.it (M.A.); sharonnatasha.cox@gmail.com (S.N.C.); brunella.ladogana@uniba.it (B.R.L.); marianna.ranieri@uniba.it (M.R.); stephanjoel.reshkin@uniba.it (S.J.R.); 2 CNR Institute of Biomembranes, Bioenergetics and Molecular Biotechnologies, National Research Council (CNR), Via G. Amendola 122/0, 70126 Bari, Italy; d.abbrescia@ibiom.cnr.it (D.I.A.); a.tullo@ibiom.cnr.it (A.T.)

**Keywords:** vasculogenic mimicry, cancer stem cells, NHE1, tumor microenvironment, acidosis, calcium signaling

## Abstract

**Highlights:**

**What are the main findings?**

- Exposure to tumor extracellular acidic pH increases cancer stem cell-mediated vasculogenic mimicry (VM) and overcomes the VM constraining induced by high collagen-containing ECMs.
- The increased VM stimulated by acidosis is paralleled by a decreased intracellular resting pHi and an increase in both NHE1 activity and an elevation in the extracellular calcium influx to increase cytosolic calcium concentration.

**What are the implications of the main findings?**

- The VM response to acidosis emerges as a key feature enabling tumor CSCs to exploit these conditions to reinforce metastatic behaviors.
- Inhibition of the activity of NHE1 represents a viable novel therapeutic strategy to block PDAC VM and reduce metastasis.

**Abstract:**

Vasculogenic mimicry (VM) is the ability of cancer stem cells (CSCs) to express an endothelial-like phenotype and participate in tumor neovascularization via the formation of a blood-conducting, matrix-rich network. We previously reported that pancreatic ductal adenocarcinoma (PDAC) CSCs develop their VM phenotype via two interacting and coordinated factors that support the formation of the VM network: (i) the overexpression of genes for endothelial factors and vascular receptors and (ii) the very high secretion of numerous pro-angiogenic/growth factors. While microenvironmental acidosis (low pHe) is an important driver of tumor metastasis, especially in PDAC, and is a component of the CSC niche, its role in VM and the ion transporters involved remains unknown. As normal stem cell differentiation is regulated by Na^+^/H^+^ exchanger 1 (NHE1)-driven pH, we investigated the role of NHE1 and the intracellular signaling involved in the acidosis-induced VM using a platform of 3D organotypic cultures composed of Matrigel with increasing concentrations of Collagen I. VM was highest on 90% Matrigel:10% Collagen I, representative of an early tumor ECM, and it decreased with increasing concentrations of Collagen I, representative of advanced tumors. In all ECM compositions, VM capacity increased stepwise with pHe acidification, and both basal and acid-stimulated VM were dependent on NHE1 activity. Acidification also decreased resting pHi and increased NHE1 proton extrusion activity, NHE1/ß1 integrin co-expression, and intracellular Ca^2+^. The stimulation of VM by extracellular acidosis depended on the transport of extracellular Ca^2+^ into the cell and the consequent increase in intracellular Ca^2+^. Altogether, these data demonstrate that extracellular acidification triggers cellular mechanisms that upregulate VM to overcome the constraints imposed by ECM composition, thereby permitting VM in ECMs where this phenotype is not expressed and extending the VM phenotype towards the tumor center to further drive metastasis.

## 1. Introduction

The tumor microenvironment (TME) is composed of stromal extracellular matrix (ECM) components, stromal cells that secrete tumor-supportive ECMs and growth factors that further alter the stromal environment [1,2,3,4,5], and in pancreatic ductal adenocarcinoma (PDAC), it can comprise up to 90% of the tumor [6]. The TME is also characterized by dynamic, interacting areas of hypoxia and acidic extracellular pH (pHe) [4,5,6,7,8,9,10]. Hypoxia is the best-characterized condition and contributes to mutagenesis, apoptosis suppression, epithelial-to-mesenchymal transition (EMT), cancer immunity, angiogenesis, and the selection of cancer stem cells (CSCs) [10,11,12,13,14,15]. It is now well established that cancer cells have an increased intracellular pH (pHi) compared to normal cells (7.4–7.6 versus 7.2), while extracellular pH (pHe) is decreased (6.4–7.0 versus 7.4). This reversal of the pH gradient in cancer cells is an early event in cancer development [16] and increases during neoplastic progression [17,18,19,20]. Extracellular acidity contributes to progression/metastasis through a combination of toxicity to adjacent normal cells, reduced immunological defenses, increased autophagy, evasion of apoptosis, metabolic adaptation, and the induction and maintenance of CSCs [19,20,21,22,23,24,25]. Tumor cells cope with acidic stress and ensure homeostasis by the expression of ion transporters/channels and enzymes such as carbonic anhydrases, Cl^−^/HCO_3_^−^ exchangers (SLC26), Na^+^/H^+^ exchangers (NHE1; SLC9A1), Na^+^/HCO_3_^−^ co-transporters (SLC4A4), monocarboxylate transporters (MCTs), gastric and non-gastric H^+^/K^+^-ATPases, etc. [20,24,25,26,27]. Importantly, NHE1-dependent extracellular acidification drives metastasis by inducing protease secretion from the invasive cancer cell structures, invadopodia [28,29,30,31,32,33].

In the last decade, it has become ever more evident that the inadequate blood perfusion typical of cancers leads to cancer cells forming a mechanism known as vasculogenic mimicry (VM). VM is the de novo formation of a perfused, matrix-rich, vasculogenic-like network of blood vessels by aggressive, usually transdifferentiated CSCs. It is now considered an alternative mechanism by which tumors organize vascular channel-like morphology to obtain nutrients without endothelial cell contribution, and VM development contributes to resistance to anti-angiogenic cancer therapies, with a strong link between the presence of VM and poor clinical outcomes [34,35,36,37,38,39,40,41,42,43,44,45,46,47]. Therefore, it is important to investigate and understand the various factors that contribute to VM and angiogenesis, including those of the TME.

CSCs produce VM in the tumor microenvironment by transdifferentiating into endothelial-like cells, lining up to form branching tubes and lumens resembling a vascular network, which provides nutrition for the tumor mass [46,47,48,49]. We have reported that CSCs derived from the PDAC cell line, Panc1, acquire a VM phenotype when grown on an early tumor ECM (Matrigel) via two interacting and coordinated factors that support the formation of the VM network: (i) the overexpression of genes for endothelial factors and vascular receptors and (ii) the very high secretion of numerous pro-angiogenic/growth factors [50]. Moreover, tumor sections derived from subcutaneous transplantation of these CSCs into nude mice exhibit increased vessel density, with a higher number of both PAS^+^ and human CD34^+^/CD31^+^ blood vessels, forming a complex mosaic vasculature that contributes to total tumor vascularization [50].

While it is now well established that hypoxia stimulates VM formation [9,15,40,43,44,49,51,52,53], whether and how acidic pHe and NHE1 activity affect VM in CSCs remains unknown. Recently, it has been reported that lowering pHi via a stiff ECM dynamically regulates VM in metastatic breast and lung cells and that experimentally increasing pHi by approximately 0.2 units was sufficient to abrogate the VM phenotype [54]. However, they did not determine the pathophysiologic mechanism driving the pHi acidification. Accordingly, in this study, we investigated how the acidic extracellular pH (pHe), a chemical signaling molecule of the metabolic microenvironment, promotes VM. We found that the ability to form VM structures was highest in 90% Matrigel:10% Collagen I and decreased as the ECM’s Collagen I content increased. In all ECM compositions, VM capacity of the CSCs increased stepwise with pHe acidification and was strongly reduced by inhibition of NHE1 activity. This occurred together with a decrease in resting pHi of approximately 0.6 units and an increase in NHE1 activity (proton extrusion capacity) of approximately 1.6-fold. Importantly, acidification did not increase CSC marker expression but did increase NHE1/ß1 integrin colocalization, which has been found to increase NHE1 activity [32]. Furthermore, the stimulation of VM formation by extracellular acidosis was strongly dependent on an increase in intracellular Ca^2+^ derived from the transport of extracellular Ca^2+^ into the cell. Altogether, our findings demonstrate that extracellular acidification facilitates VM by triggering cellular mechanisms that upregulate VM to overcome the restrictions of the ECM composition on VM and extend the vascular phenotype to the typical fibrotic ECMs of the more advanced tumor to further drive metastasis [50].

## 2. Materials and Methods

### 2.1. Cell Line

Experiments were performed on cancer stem cells (CSCs) selected from the PDAC cell line Panc-1, identified by their ability to form anchorage-independent colonies and by their overexpression of common CSC markers, as previously described [50,55,56]. The CSCs were cultured in CSC medium, i.e., Dulbecco’s modified Eagle’s medium (DMEM)/F-12, without glucose (US biological Life Sciences, Rome, Italy) supplemented with 1 gm·L^−1^ glucose, B27 serum substitute (Gibco, Life Technologies, Monza, Italy), 1 μg/mL Fungizone (Gibco, Life Technologies), 1% penicillin/streptomycin (Gibco, Life Technologies), 5 μg/mL heparin (Sigma-Aldrich, Darmstadt, Germany), 20 ng/mL growth factors (EGF (Peprotech), and 20 ng/mL FGF, both from Peprotech, Rocky Hill, NJ, USA) at 37 °C in a 5% CO_2_ atmosphere.

### 2.2. Acidic Low pH Medium

For pH adjustments of the cell culture media to either pHe 7.0 or 6.7, the CSC medium was supplemented with NaHCO_3_ according to the Henderson–Hasselbalch equation to achieve the target pH_e_. The medium osmolarity was balanced with NaCl. The initial and final pH were measured using a WTW InoLab Benchtop pH meter. The different powder components were dissolved in UltraPure distilled water (Invitrogen, Monza, Italy; Cat# 10977-035), and the resulting medium was filtered under sterile conditions and supplemented with B27 and all the components of the CSC medium reported above.

### 2.3. Organotypic 3D Culture

3D organotypic cultures were prepared using Matrigel Basement Membrane Matrix (Corning, Pisa, Italy) and Collagen I (bovine-Gibco, Life Technologies), as previously described [50,56]. Matrigel was thawed on ice and diluted to a final concentration of 7 mg/mL in serum-free media, whereas Collagen I was diluted to a final concentration of 3 mg/mL in distilled, sterile water, PBS 10× (Sigma-Aldrich, Darmstadt, Germany), and 0.015 N NaOH.

From these initial concentrations, three different Matrigel/Collagen I mixtures at defined final concentrations (90% M–10% C, 80% M–20% C, 70% M–30% C) were obtained.

Each mixture (100 μL) was polymerized in 96-well plates at 37 °C for 60 min, and then cells were seeded at 15,000 cells/well in either neutral (pH 7.4) or acidic (pH 6.7) DMEM-F12 medium on top of the gels. Treatments were added 30 min after seeding to allow cell attachment.

### 2.4. Intracellular Calcium Determination

For intracellular calcium concentration [Ca^2+^]i measurements, cells were grown on Ø25 mm glass coverslips. Cells were loaded with 4 µM Fura-2 AM for 30 min at 37 °C in DMEM-F12. Ringer’s Solution was used to perfuse cells during the experiment, containing 20 mM Hepes, 0.8 mM MgSO_4_, 3 mM KCl, 1.8 mM CaCl_2_, 135 mM NaCl, 11 mM glucose, and 1 mM KH_2_PO_4_ at pH 7.4. Measurements were performed using an inverted microscope (Nikon Eclipse TE2000-S, Nikon Europe B.V., Amstelveen, The Netherlands) equipped for single-cell fluorescence measurements and analysis. The sample was illuminated with a 40× oil-immersion objective (NA = 1.30). The Fura-2 AM-loaded sample was excited at 340 and 380 nm using the FuraLED light source for Ratiometric Calcium Imaging at 3 s intervals (CAIRN Research, Rapp OptoElectronic GmbH, Wedel, Germany). Emitted fluorescence was passed through a dichroic mirror, filtered at 510 nm (Omega Optical, Brattleboro, VT, USA), and captured by a cooled CCD camera (CoolSNAP HQ, Photometrics, Leica Microsystems Srl, Milan, Italy). Fluorescence measurements were carried out using Metafluor software version 7.8.1.0 (Molecular Devices, MDS Analytical Technologies, Toronto, ON, Canada). Cells were incubated at pH 7.4 or pH 6.7 for 24 h, and intracellular calcium levels were then measured. Intracellular calcium level was calibrated and then calculated as described by Grynkiewicz [57]. Briefly, calcium concentration was determined from the emission fluorescence ratio of the two excitation wavelengths accordingly to the formula [Ca^2+^]i = Kd × Q(R − Rmin)/(Rmax − R), where Kd (224 nM) indicates the dissociation constant of Fura-2 AM for [Ca^2+^]i and Q indicates the ratio of the fluorescence intensities (F) at the minimum and the maximum calcium concentration at 380 nm. Each sample was calibrated by adding 5 µM ionomycin in the presence of 1 mM EGTA, without calcium.

### 2.5. Intracellular pH and NHE1 Activity Determination

We evaluated resting pHi and NHE1 activity in the cells by monitoring intracellular pH (pHi) changes in BCECF-loaded cells as previously described [58]. Cells were loaded with 2 µM BCECF-AM (2′,7′-Bis-(2-Carboxyethyl)-5-(and-6)-Carboxyfluorescein-Acetoxymethyl Ester) for 30 min at 37 °C in DMEM-F12.2, and intracellular pHi was determined by calculating the ratio of BCECF emission intensity at 535 nm. The dye was excited at ~490 nm, and its isosbestic point was set at ~440 nm. We calibrated the 490/440 BCECF fluorescence ratio by incubating the cells in a high K^+^ solution (140 mM KCl, 4.6 mM NaCl, 1 mM MgCl2, 2 mM CaCl_2_, 10 mM Hepes, 5 mM glucose), followed by permeabilization with 5 mM nigericin to equilibrate extracellular pH (pHo) with pHi. We then adjusted the pH of the bathing solution between 6.6 and 8.5. The 490/440 ratio remained linear within this pH range (r = 0.96, *n* = 6). NHE1 activity was measured by monitoring pHi recovery after an acid load produced with the NH_4_Cl pre-pulse technique. The pHi dependence of intrinsic buffering capacity (ßi), i.e., the buffering power of all non-HCO_3_-CO_2_ buffers, was measured by the NH_4_ pulse method and calculated as follows: ßi = [NH_4_]/pHi. The actual activity of the NHE1 in terms of proton flux rate (mM H^+^/min) is determined by multiplying the rate in pHi change by cells ßi: V_H+_ (mM/min) = ßi (mM/pHi) × pHi/t (pHi/min).

### 2.6. Gel Electrophoresis and Immunoblotting

CSC cells lysates were separated on 7.5% stain-free polyacrylamide gels (Bio-Rad Laboratories, Inc., Hercules, CA, USA) under reducing conditions. Protein bands were electrophoretically transferred onto Immobilon-P membranes (Millipore Corporate Headquarters, Billerica, MA, USA) for Western blot analysis, blocked in EveryBlot blocking buffer (Bio-Rad Laboratories, Milan, Italy), and incubated with primary antibodies overnight. Primary antibodies included monoclonal integrin ß1 (Santa Cruz (K-20), 1:100 in 3% BSA), monoclonal Nestin 10C2 (Cell Signaling Technology, London, UK #33475, 1:1000 in 3% BSA), monoclonal ALDH1A1 B5 (Santa Cruz (B-5), 374149, 1:50 in 3% BSA), and polyclonal anti-NHE1 (Alomone Labs, Milan, Italy ANX-010), 1:25 in 3% BSA). Immunoreactive bands were detected with secondary antibodies conjugated to horseradish peroxidase (HRP), obtained from Santa Cruz Biotechnology (Tebu-Bio, Milan, Italy). Bands were visualized using Clarity™ western ECL substrate on a ChemiDoc system (Bio-Rad Laboratories, Milan, Italy), normalized to total protein via stain-free technology, and quantified by densitometry with Image Lab (Bio-Rad Laboratories, Milan, Italy); statistical data analysis was performed in GraphPad Prism v6.01 (GraphPad Software, San Diego, CA, USA).

### 2.7. Vascular Network Analysis

To quantify vascular parameters, CSCs were cultured as above for 24 h under these growth conditions. Vascular channel networks were photographed using the TE200 microscope (Nikon USA, Garden City, NY, USA), and the development of capillary-like structures (VM) was analyzed as previously described [50]. To analyze the role of intracellular Ca^2+^ homeostasis/influx, cells were treated with 10 µM BAPTA-AM (1,2-Bis(2-aminophenoxy)ethane-tetraacetic acid tetrakis(acetoxymethyl ester) or increasing concentrations of EGTA (ethylene glycol-bis(β-aminoethyl ether)-N,N,N′,N′-tetraacetic acid) (0.2, 0.5, 1, 2 mM), respectively, 30 min after cell seeding, and they were incubated overnight at pH 7.4 or 6.7.

### 2.8. Immunofluorescence

A 24-well cell culture plate was prepared by placing a 12 mm diameter glass in each well. Matrigel and Collagen I were prepared as reported above. After matrix polymerization, 1.5 × 10^4^ cells/well were seeded. On the 5th day, an IF assay was performed as follows. Primary antibodies, monoclonal integrin ß1 (Santa Cruz (K-20), 1:100 in 3% BSA), and polyclonal anti-NHE1 (Alomone Labs, ANX-010), 1:25 in 3% BSA) were incubated overnight at 4 °C. The next day, cells were washed three times for 5 min with 1% BSA and incubated for 45 min at room temperature with either goat anti-mouse 568 (1:1000 in 1% BSA) or goat anti-rabbit 488 (1:1000 in 1% BSA), both from Invitrogen. Samples were mounted using Fluoroshield mounting medium (Sigma, Cat# F6182) and imaged on a Nikon TE 2000S epifluorescence microscope equipped with MicroMax 512BFT CCD camera (Princeton Instruments, Trenton, NJ, USA) and a Nikon lamp shutter, using a mercury short-arc photo-optic HBO 103 W/2 lamp for excitation (OSRAM GmbH, Augsburg, Germany). Colocalization between the two fluorescent signals was quantified using NIS-Elements AR software version 6.20.00 (Nikon Instruments) by calculating Pearson’s correlation coefficient, which measures the linear relationship between signal intensities from −1 (negative correlation) to +1 (positive correlation).

### 2.9. RNA-Seq and Data Analysis

Total RNA was extracted from cells using the RNeasy Mini Kit Qiagen, according to the manufacturer’s instructions. RNA integrity was assessed using an Agilent 2100 Bioanalyzer with the RNA6000 Pico Chip (Agilent Technologies, Santa Clara, CA, USA), and RNA concentration was assessed using a Qubit RNA High Sensitivity (Life Technologies). Directional RNA-seq libraries were prepared from 100 ng of total RNA using the Illumina Stranded Total RNA Prep, Ligation with Ribo-Zero Plus, following the manufacturer’s protocol. Sequencing was performed on an Illumina NovaSeq 6000 (Illumina, San Diego, CA, USA).

### 2.10. Statistical Analyses

Statistical differences between experimental groups were evaluated using unpaired *t*-tests with Welch’s correction or one-way ANOVA followed by Tukey’s post-test in GraphPad Prism v6.01. The results are presented as mean ± SEM, and differences were considered statistically significant at *p* < 0.05.

## 3. Results

Acidic extracellular pH (pHe) increases the formation of vasculogenic mimicry (VM) of PDAC CSCs on Matrigel-rich ECM and activates the VM program on more fibrotic collagen I-rich ECMs that normally do not produce VM structures.

**Figure 1:**
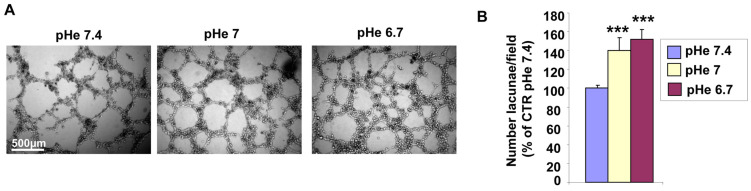
Effect of acidic extracellular pH (pHe) on VM formation. (**A**) Typical images of the formation of vasculogenic mimicry of CSCs on 90% Matrigel:10% Collagen I at three decreasing pHes. (**B**) Histogram of the number of lacunae per field (completely closed areas within the tube-like structures). *n* = 5 independent experiments. *p* < 0.001 (***) versus pHe 7.4.

We have previously shown that Panc1-derived CSCs produce vasculogenic mimicry (VM) in vitro when cultured on Matrigel-rich ECMs at pH 7.4 and in vivo in subcutaneous tumors [50], and that co-incubation of these CSCs with cancer-activated fibroblast-derived tumor-conditioned medium stimulates VM [56]. To assess the effects of acidic pHe on VM of these CSCs, we established acidic culture media to maintain pHe’s of 7.4, 7.0, and 6.7 at 37 °C in 5% CO_2_ (see M&M). CSCs were seeded in the 3D organotypic setup consisting of a mix of 90% Matrigel:10% Collagen I, with culture medium at pH 7.4, pH 7.0, and pH 6.7 for 24 h, at which time the level of VM was measured as described in the M&M. Figure 1A displays typical images showing that cell incubation in acidic pHe stepwise increased the organization of the VM structures resulting in a significant increase in the mean number of lacunae as pHe becomes more acidic (Figure 1B).

**Figure 2:**
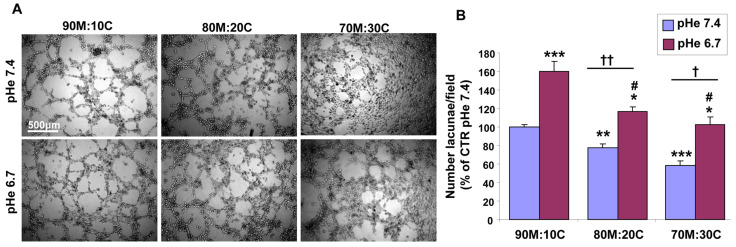
Effect of ECM composition on acidic pHe-stimulated VM formation. (**A**) Representative images of the formation of vasculogenic mimicry of CSCs on three ECM mixes and at two pHes. (**B**) Histogram of the quantification of the number of lacunae per field. *n* = 5 independent experiments. *p* < 0.05 (*), *p* < 0.01 (**) or *p* < 0.001 (***) versus 90M:10C pHe 7.4; *p* < 0.05 (†), *p* < 0.01 (††), and pHe 6.7 versus pHe 7.4 on their respective ECM; *p* < 0.05 (#) and *p* < 0.01 versus 90M:10C pHe 7.4.

As we previously showed that at pHe 7.4 the CSCs no longer form VM structures as the concentration of Collagen I increases in the ECM [50], we next investigated whether low pHe could turn on the VM program in cells in ECMs that do not usually support it. For this, the CSCs were cultured on a platform of different ECM mixtures with increasing concentrations of Collagen I (90% Matrigel:10% Collagen I; 80% Matrigel:20% Collagen I; and 70% Matrigel:30% Collagen I) at either 7.4 or 6.7 for 24 h. While increasing ECM Collagen I concentrations reduced VM formation at pHe 7.4, incubation at pHe 6.7 promoted VM compared to the cells grown on the same ECMs at pHe 7.4, such that the mean number of lacunae on a mix of 80% Matrigel:20% Collagen I and 70% Matrigel:30% Collagen I at pHe 6.7 significantly increased compared to that of pHe 7.4. Interestingly, the mean number of lacunae on 80% Matrigel:20% Collagen I and 70% Matrigel:30% Collagen I at pHe 6.7 also significantly increased compared to the number of lacunae on the 90% Matrigel:10% Collagen I ECM at pHe 7.4 (Figure 2A,B).

Altogether, these data demonstrate that acidic pHe triggers molecular pathways that activate the VM phenotype also in ECMs where this phenotype is not expressed. As the tumor center has higher Collagen I levels, this would extend the VM phenotype toward the tumor center, further expediting tumor dissemination [50].

### 3.1. Collagen I-Rich ECM Increases While Acidic Extracellular pH (pHe) Decreases the Expression of CSC Stemness Markers

**Figure 3:**
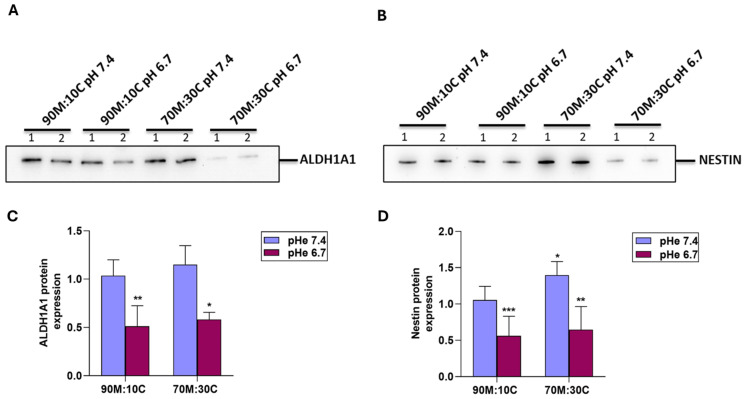
Effect of acidic extracellular pH (pHe) on CSC marker expression on different ECM compositions. Analysis of ALDH1A1 and Nestin protein expression in cells cultured on 90M:10C or 70M:30C matrices at pHe 7.4 or 6.7. Representative Western blots (**A**,**C**) and corresponding quantification (**B**,**D**) show that protein levels of ALDH1A1 and Nestin decrease under pHe 6.7 conditions. In contrast, both markers, particularly Nestin, show a marked increase when cells are cultured on a collagen-rich matrix. Statistical analysis was performed using two-way ANOVA; *p* < 0.05 (*), *p* < 0.01 (**), and *p* < 0.001 (***) compared to 90M/10C at pH 7.4.

One possible explanation for the acidic pHe stimulation of VM is that low pHe promotes could modulate the CSC stemness profile, as has been reported for hypoxia [53] or Wnt5a expression [59]. For this, we incubated the CSCs as above for 3 days and measured the expression of two known PDAC CSC markers, Nestin and ALDH1A1 [60], by Western blot. Figure 3 shows that cells growing in 70% Matrigel:30% Collagen I ECM had higher CSC marker expression levels than those on 90% Matrigel:10% Collagen I, and their growth at pHe 6.7 for 3 days decreased the expression of Nestin and ALDH1A1. The levels of Nestin and ALDH1A1 in each of these growth conditions were also determined by RNA sequence analysis and confirmed the same trends (Supplemental Figure S1). This suggests that while both ECM composition and acidic pHe modify PDAC CSC stemness in a complex manner, changes in stemness do not play a role in either ECM-dependent or acidosis-dependent VM. Therefore, it is necessary to determine the transporter and signal transduction systems involved in this phenomenon.

### 3.2. Acidic Extracellular pH (pHe) Reduces Intracellular Resting pH (pHi) and Increases NHE1 Proton Extrusion Activity

**Figure 4:**
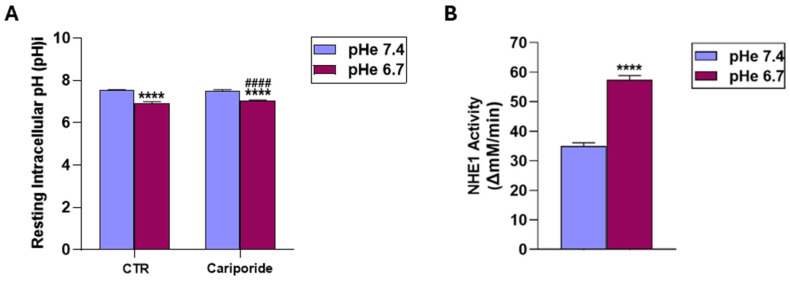
Effects of extracellular pH on resting intracellular pH and NHE1 activity. (**A**) Resting intracellular pH (pH)i in cells treated or not with cariporide and exposed to pHe 7.4 or pHe 6.7. *p* < 0.0001 (****) resting pHi at pHe 6.7 compared to their respective pHe 7.4 and *p* < 0.0001 (####) resting pHi compared to pHe 7.4 with cariporide. (**B**) NHE1 proton extrusion activity (ΔmM/min), calculated by multiplying the rate of NHE1-dependent pHi change with the corresponding intracellular buffering capacity (ßi), measured at pHe 7.4 and pHe 6.7, as described in Materials and Methods. NHE1 proton extrusion activity is significantly increased under acidic conditions. *p* < 0.0001 (****) NHE1 proton extrusion activity at pHe 6.7 compared to NHE1 proton extrusion activity pHe 7.4.

Among the ion transporters involved in pHi regulation, the Na^+^/H^+^ exchanger isoform 1 (NHE1) also regulates proliferation, differentiation, and epithelial morphogenesis [61,62,63], and its inhibition has been found to inhibit angiogenesis and the expression/secretion of pro-angiogenic factors [64]. We next determined the effect of incubation at pHe 6.7 for 24 h on the resting intracellular pH (pHi), on the buffering capacity of the cells and on NHE1 proton extrusion activity (∆mM H^+^/min) using the specific, selective, and potent NHE1 inhibitor, cariporide (HOE642) [64,65,66,67,68,69], at 5 μM for 24 h. Buffering capacity (ßi) increased from 128 +/− 19.2 to 282 +/− 40.8 mM/pHi at pHe 7.4 and 6.7, respectively. The effect of cariporide treatment on recovery after an acid load and the resulting NHE1 activity as ∆pHi/min are shown in Supplemental Figure S2. As can be seen in Figure 4A, exposure to pHe for 24 h lowered resting pHi by 0.6 units (7.53 +/− 0.03 vs. 6.93 +/− 0.66 at pHe 7.4 vs. 6.7, respectively) and increased the NHE1 proton extrusion capacity (Figure 4B) by approximately 1.6-fold (35.1 +/− 1.02 vs. 57.4 +/− 1.43 nM H^+^/min at pHe 7.4 vs. 6.7, respectively). The effect of cariporide treatment on recovery after an acid load and the resulting NHE1 activity as ∆pHi/min are shown in Supplemental Figure S2.

### 3.3. NHE1 Activity Is Necessary for Basal and Acidic Stimulation of VM

**Figure 5:**
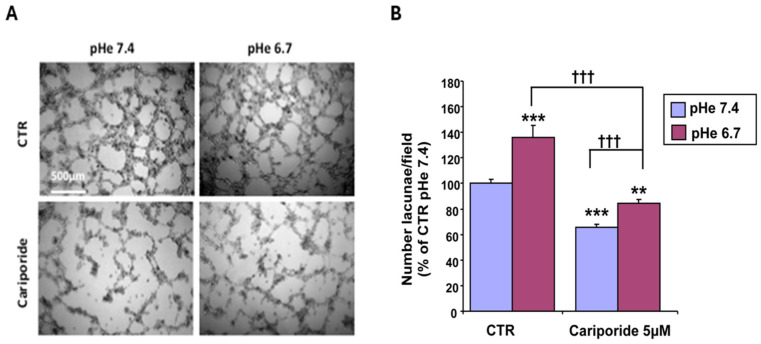
Inhibition of NHE1 reduces both basal and acidosis-stimulated VM. (**A**) Typical images of the formation of vasculogenic mimicry of CSCs on 90M:10C and at two pHes. (**B**) Histogram of the number of lacunae per field; *n* = 5 independent experiments. *p* < 0.01 (**) or *p* < 0.001 (***) versus pHe 7.4 CTR; *p* < 0.001 (†††) cariporide pHe 6.7 versus either cariporide pHe 7.4 or CTR pHe 6.7.

To functionally determine the role of NHE1 in the basal capacity of CSCs to form an acidity-promoted VM network, we incubated the CSCs on Matrigel with 5 μM cariporide for 24 h. As shown in Figure 5A, cariporide greatly reduced VM complexity at both physiological pH and in an acidic pH medium, as the mean number of closed lacunae decreased sharply (Figure 5B).

**Figure 6:**
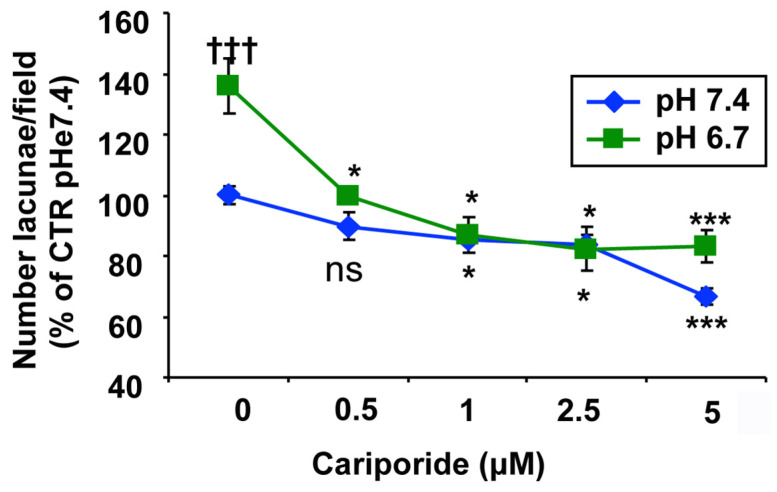
Dose–response curve of cariporide concentration on VM formation on 90M:10C ECM at pH 7.4 (blue curve) vs. pH 6.7 (green curve); *n* = 5 independent experiments. ns = non significant, *p* < 0.05 (*) or *p* < 0.001 (***) versus their respective control values; 90M:10C pHe7.4; *p* < 0.001 (†††) CTR pHe 6.7 versus CTR pHe 7.4.

We next performed dose-response measurements with cariporide to determine if its inhibitory dynamics on VM differ between physiological and acidic pHe. As shown in Figure 6, the inhibition of the VM structures by cariporide was much more evident at pHe 6.7 than at 7.4. This is consistent with the reported higher binding affinity of cariporide at more acidic pHe [66,67,69] and the general inhibitor dynamics for more active enzymes/transporters [70].

**Figure 7:**
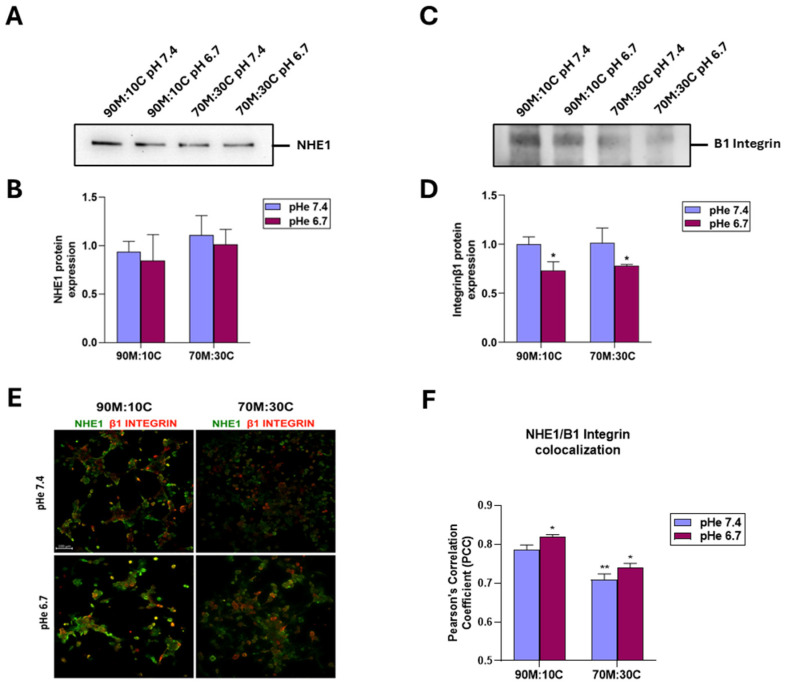
Combined analysis of NHE1 and β1-integrin protein expression in cells cultured on 90M:10C or 70M:30C matrices at pH 7.4 or 6.7. Representative Western blots (**A**,**C**) and corresponding quantifications (**B**,**D**) show stable NHE1 levels and a significant reduction in β1 integrin under acidic conditions. Statistical analysis was performed using two-way ANOVA; *p* < 0.05 (*) compared to 90M/10C at pH 7.4. (**E**) Effect of acidic pHe on NHE1 and ß1 integrin expression/colocalization. Typical images of NHE1 (green) and ß1 integrin (red) expression on either 90% Matrigel:10% Collagen I (90M:10C) or 70% Matrigel:30% Collagen I (70M:30C) at either pHe 7.4 or pHe 6.7. Immunofluorescence was performed as described in Materials and Methods. Scale bar = 100 µM. (**F**) Histogram showing Pearson’s correlation coefficient (PCC) values from colocalization analysis. *p* < 0.05 (*) and *p* < 0.01 (**) compared to 90M/10C pH 7.4.

As the ß1 integrin is known to play a role in VM formation [37] and to interact with NHE1 [32], we next determined whether acidic pHe could modify VM formation by altering NHE1 or ß1 integrin expression and/or colocalization. As shown in the typical images in Figure 7, NHE1 expression remains unchanged regardless of ECM composition or extracellular acidosis, while ß1 integrin expression is consistently reduced at acidic pHe and unaffected by ECM composition. Despite this, the colocalization of NHE1 and ß1 integrin is reduced in 70% Matrigel:30% Collagen I and increased in extracellular acidosis.

### 3.4. Effect of Acidic pHe on Intracellular Calcium and Its Source and Role of Intracellular Calcium in the Acidic pHe Stimulation of VM

It is increasingly evident that Ca^2+^ is involved in a wide range of cancers, with dysregulated Ca^2+^ homeostasis playing a key role in carcinogenesis and tumorigenesis [71,72,73], and there is an interplay between Ca^2+^ signaling and TME hypoxia and acidosis [74]. In this respect, it was demonstrated that intracellular and extracellular Ca^2+^ levels regulate the formation of a vasculogenic-like network of aggressive melanoma cells at physiological pHe [75,76].

**Figure 8:**
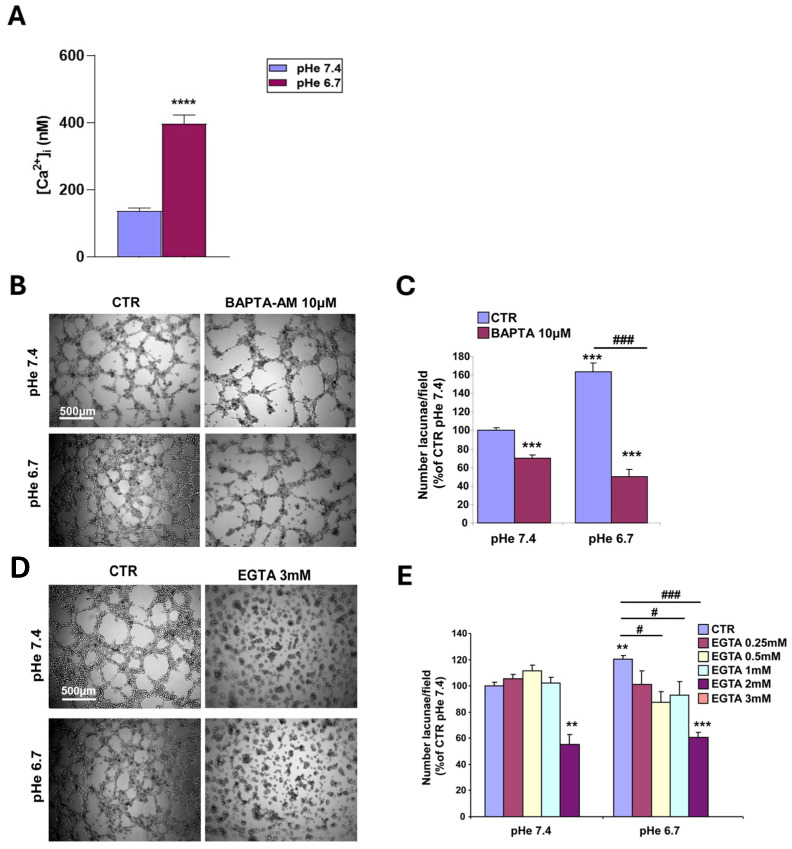
Role of intracellular and extracellular calcium (Ca^2+^) in the regulation of VM at pH 7.4 and 6.7. (**A**) Intracellular Ca^2+^ levels were measured in cells grown on 90% Matrigel:10% Collagen I at pH 7.4 and 6.7. Acidic pH significantly increases [Ca^2+^]_i_. (**B**,**C**) Intracellular Ca^2+^ was buffered by incubating the cells with 10 µM BAPTA-AM as described. (**D**,**E**) Extracellular Ca^2+^ was titrated with increasing concentration of the calcium chelator EGTA in the medium. (**B**,**D**) Typical images of BAPTA-AM and 3 mM EGTA, respectively. (**C**,**E**) Histograms of the number of lacunae per field. *n* = 5 independent experiments. *p* < 0.01 (**) *p* < 0.001 (***) or *p* < 0.001 (****) versus control (CTR) pHe7.4; *p* < 0.05 (#) and *p* < 0.001 (###) versus CTR pHe 6.7.

Therefore, we next determined whether acidic pHe stimulates the VM phenotype by altering intracellular Ca^2+^ homeostasis and whether this effect depends on Ca^2+^ influx. We first measured intracellular Ca^2+^ concentration in a population of single cells after a 24 h incubation at pHe 6.7, as described in the Materials and Methods. Figure 8A shows that incubation at pHe 6.7 for 24 h increased intracellular Ca^2+^ (from 137.4 +/*−* 7.8 to 396.6 +/*−* 26.5 nM). Pre-treatment of cells with 10 μM of the intracellular calcium buffer, BAPTA-AM, produced a net reduction in the VM tubular network both at physiological pHe and at acid pHe compared to their respective controls (Figure 8B,C). However, the cells were much more sensitive to this treatment at acidic pHe (32% +/*−* 5.7 vs. 69% +/*−* 6.6 inhibition at 7.4 and 6.7, respectively; *p* < 0.01). This may be due to increased Ca^2+^ influx at lower pHe. Therefore, we next tested this hypothesis by incubating the cells with increasing concentrations of the extracellular Ca^2+^ chelator, EGTA. Indeed, while extracellular Ca^2+^ removal by 3 mM EGTA completely abolished VM formation at both pHes, only the cells at pHe 6.7 were sensitive to lower EGTA concentrations (Figure 8D,E), in line with an acid-dependent stimulation of calcium influx. These data suggest that the transport of extracellular Ca^2+^ into the cytosol regulates the VM phenotype and that VM stimulation by the acidic TME occurs through a transport-dependent increase in intracellular Ca^2+^ levels from the extracellular medium. The calcium channels involved in this increase in intracellular Ca^2+^ will be the subject of further studies.

## 4. Discussion

While great advances have been made in treating cancer, with a decline in the past 30 years of overall cancer mortality, this progress is met with little effect once the disease spreads beyond the primary site. Indeed, the turning point in cancer progression is metastasis, wherein cancer spreads within the body as individual cells detach from the primary tumor and travel to distant body regions via the bloodstream or lymphatic system. This spread to distant organs is a very complex multi-step process in which cancer cells invade surrounding tissues at the primary site, intravasate, survive in the circulation as circulating tumor cells, and extravasate at distant organs to form metastases. There are a few treatment options for metastatic disease, which is responsible for more than 90% of the nearly 10 million cancer deaths globally each year [77,78,79,80,81,82,83,84].

One important common characteristic of many solid tumors is their abnormal, inefficient vasculature, which includes both blood and lymphatic vessels, and this feature contributes to a microenvironment characterized by low pHe, hypoxia, metabolic reprogramming, and immune evasion [15,85,86]. To overcome this challenge, tumors use neo-angiogenesis to support tumor growth and metastasis. The tumor cells guide the assembly of abnormal blood vessels to access oxygen and nutrients, recruit protumor and suppress antitumor immune cells, and educate fibroblasts for extracellular matrix remodeling and growth factor secretion [85]. Angiogenesis is one of the principal mechanisms by which cancer cells have sufficient blood supply and nutrition for their growth demands [87,88], and very few tumors can grow without angiogenesis even in hypoxic conditions [89]. Therefore, it was expected that anti-angiogenic drugs could improve the outcome of chemotherapy and patient survival, and inhibiting angiogenesis and/or normalizing the tumor vasculature are widely used in the treatment of cancer patients. However, the clinical results have been disappointing [89,90,91].

In this context, tumor vascularization can also occur by vasculogenic mimicry (VM), a novel and functional tumor microcirculation without the contribution of endothelial cells, which is an alternative survival strategy adopted by cancer cells under hypoxic stress that allows them to adapt, grow, and disseminate, which is significantly related to poor prognosis and adverse clinico-pathological parameters [36,37,38,39,40,41,42,44,46]. Indeed, the metastatic process starts when tumor cells acquire two specific functional abilities, invadopodia-mediated invasion and VM [50,84].

Tumor cell plasticity underlies VM, as aggressive tumor cells revert to an undifferentiated stem cell-like phenotype, suggesting that the cancer stem cell (CSC) subpopulation in the tumor organizes VM in response to environmental conditions. Indeed, the cancer cells able to produce VM display multipotent, stem cell-like phenotypes, suggesting a remarkable degree of plasticity and, mechanistically, VM biogenesis is closely linked to EMT and CSCs [50,53]. It has been demonstrated that CSCs promote VM in the TME by differentiating or trans-differentiating into endothelial-like cells [92], lining up to form branching tubes and lumens resembling a vascular network, which provides nutrition for cancer cells [93]. Furthermore, CSC markers are also correlated with in vivo VM, since the cells that contribute to VM are characterized by the expression of CD133, aldehyde dehydrogenase 1 (ALDH1A1), Nestin, and CD44 [50,52,59,94]. However, we observed that while both ECM composition and acidic pH modify CSC stemness in a complex manner (Figure 3), this does not play a role in either ECM-dependent or acidosis-driven VM. Therefore, it was necessary to determine the transporter and signal transduction systems involved in this phenomenon.

While the role of the hypoxic TME in driving VM is well documented [9,15,40,43,44,49,51,52,53], other drivers may also initiate the VM process, including anti-angiogenic therapies and microenvironment-derived molecules [9,91,95,96]. However, to date, relatively little is known at the molecular level about tumor cells’ ability to form highly patterned VM channels. Here, we demonstrate that VM is also upregulated by the acidic component of the TME, as VM capacity increased in a stepwise manner with pHe acidification (Figure 1). This occurred in all ECM compositions (Figure 2), and both basal and stimulated VM were dependent on NHE1 activity (Figure 5 and Figure 6). Extracellular acidification decreased intercellular pH (pHi) by approximately 0.6 pH units and increased NHE1 proton extrusion activity by approximately 1.6-fold via an increase in cellular buffering capacity (Figure 4). These results are consistent with recent observations that an acidified pHi is both necessary and sufficient for VM in breast and lung cancer cells [54]. Further, the acidic stimulation of VM was mediated by an increase in intracellular Ca^2+^ resulting from extracellular influx (Figure 8). Therefore, in addition to the intrinsic regulation of VM by hypoxia and ECM composition, VM is also upregulated by the acidic TME via NHE1 activity, necessitating increased intracellular Ca^2+^ derived from extracellular Ca^2+^ influx. Importantly, extracellular acidosis stimulated VM, even in ECMs that normally do not support it. By activating cellular mechanisms that upregulate VM and overcome ECM constraints, acidity may extend VM deeper into the collagen-enriched tumor center, thereby enhancing metastasis. This CSC-derived vasculogenic network, combined with acidosis-driven local invasion [34], suggests that the acidic TME specifically activates the previously described symbiotic relationship between the parenchymal PDAC tumor cells and the CSCs that underlie early PDAC infiltration and metastasis [50].

While the potential stimulation of VM by acidosis has recently been proposed in melanoma [9] and lung cancer [97], our study is, to our knowledge, the first to provide evidence that low extracellular pH drives VM, and that this occurs through NHE1 activity and calcium influx signaling. These results provide new insights into the role of the extracellular pH in cancer metastasis and point to potential therapeutic avenues. In this regard, cariporide (HOE642) has been approved for clinical use in cardiac ischemia perfusion-reperfusion injury due to its protective effects [66] and has been proposed for use in the cancer setting [67,68,69]. Since one of the causes of the limited efficacy of anti-angiogenic drugs is that they induce hypoxia, which, in turn, promotes the VM formation, the inhibition of NHE1 represents a potential novel therapeutic strategy for blocking both endothelial cell- and CSC-driven neovascularization.

## 5. Conclusions

In conclusion, here we demonstrate that exposure to tumor extracellar acidic pH increases PDAC cancer stem cell-mediated vasculogenic mimicry (VM) in all ECM compositions. This increases our understanding of the mechanisms by which the acidic stromal tumor microenvironment modulates the malignant progression of pancreatic ductal adenocarcinoma (PDAC), highlighting the dynamic interplay among microenvironmental factors and cellular plasticity. Importantly, this occurs via an acidification of intracellular pH through the stimulation of NHE1 activity and an increase in cytosolic calcium concentration via calcium influx. Notably, acidosis enables VM to occur even in ECM contexts that are normally non-permissive (i.e., collagen I-rich ECMs), potentially extending this phenotype toward the tumor core and further promoting metastatic progression. The identification of NHE1 as a regulator of VM validated the use of its specific inhibitor, cariporide, and opens new therapeutic perspectives, including future combinatorial approaches of cariporide with already utilized therapies, such as Gemcitabine and/or nab-paclitaxel. Future studies will be conducted to determine both the underlying mechanisms of the complex interactions between NHE1, ß1 integrin, and calcium mobilization and the pH-dependent calcium channels that are responsible for the calcium influx. In summary, the continuation of this research will enable the translation of mechanistic insights into clinically relevant applications, ultimately improving therapeutic strategies and patient outcomes in pancreatic cancer.

## Data Availability

The original contributions presented in this study are included in the article/Supplementary Material. Further inquiries can be directed to the corresponding authors.

## Supplementary Materials

The following supporting information can be downloaded at: https://www.mdpi.com/article/10.3390/cells15100865/s1; Figure S1: RNA-seq analysis of ALDH1A1 (A) and Nestin (B) transcript levels; Figure S2: Effects of NHE1 inhibition by Cariporide on intracellular pH recovery and NHE1 activity under physiological and acidic conditions.
