# Supplementary material for: Acidosis Drives Vasculogenic Mimicry in PDAC CSCs via Na+/H+ Exchanger Isoform 1 (NHE1) and Calcium Entry
Source: Cells. 2026 May 9;15(10):865. doi: 10.3390/cells15100865 (PMC13204406; doi:10.3390/cells15100865)
Supplement: Supplementary file 1 [file cells-15-00865-s001.zip › cells-4223560-supplementary.pdf]

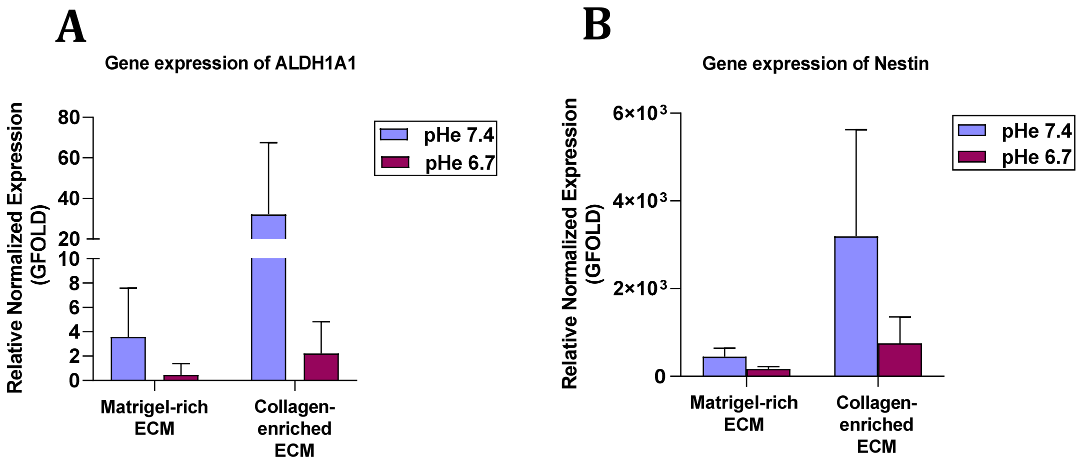

Supplemental Figure S1. RNA-seq analysis of ALDH1A1 (A) and Nestin (B) transcript levels in cells cultured on Matrigel-rich or collagen-rich matrices under physiological (pHe 7.4) or acidic (pHe 6.7) conditions. Expression values represent GFOLD library size-normalized counts derived from four independent experiments. Both transcripts show higher expression in collagen-rich matrices, with reduced levels at acidic pHe.

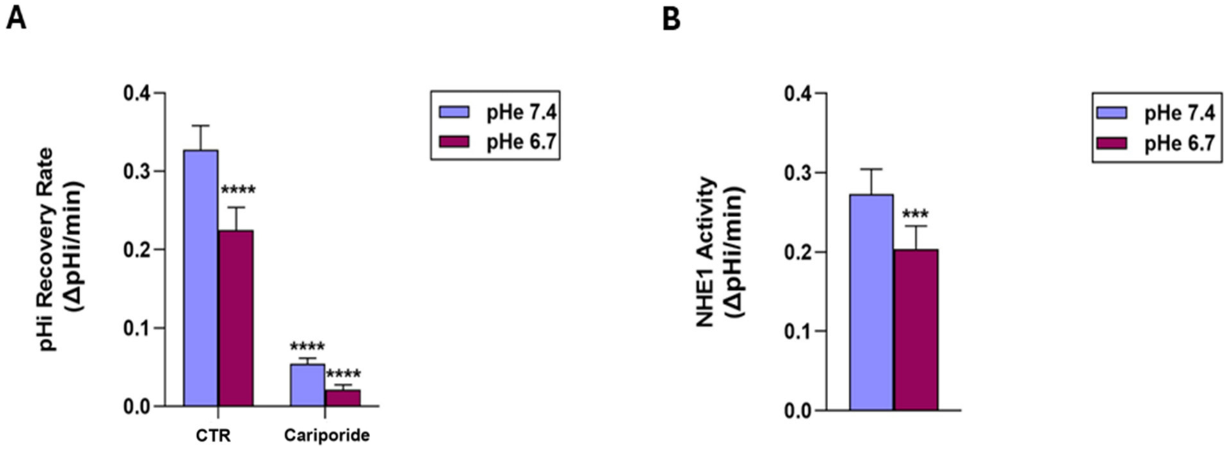

Supplemental Figure S2. Effects of NHE1 inhibition by Cariporide on intracellular pH recovery and NHE1 activity under physiological and acidic conditions. (A) Intracellular pH recovery rate **(**ΔpH/min), in cells treated or not with Cariporide and exposed to pH 7.4 or pH 6.7. Pharmacological inhibition of NHE1 markedly reduces pH recovery, with an additional decrease under acidic conditions. **(B)** NHE1 activity (ΔpH/min) measured at pHe 7.4 and pHe 6.7, showing a pronounced reduction in acidic medium. Statistical significance is indicated as ***$p<0.001$, ****$p<0.0001$.
